# Development and validation of the Iranian version of the Children's Experiences of Dental Anxiety Measure (CEDAM)

**DOI:** 10.1002/cre2.830

**Published:** 2024-01-18

**Authors:** Zahra Enshaei, Kimia Sadeghi Kaji, Zahra Saied‐Moallemi

**Affiliations:** ^1^ Department of Pediatric Dentistry, Dental Research Center, Dental School Isfahan University of Medical Sciences Isfahan Iran; ^2^ Dental School Isfahan University of Medical Sciences Isfahan Iran; ^3^ Department of Oral Public Health, Dental School, Dental Research Center Isfahan University of Medical Sciences Isfahan Iran

**Keywords:** child, dental anxiety, questionnaire, validity

## Abstract

**Objective:**

The aim of this study was to develop the Iranian version of the Children's Experiences of Dental Anxiety Measure (CEDAM) and evaluate its validity and reliability in assessing dental anxiety in children aged 9‒16.

**Methods:**

The CEDAM was translated into Persian following the guidelines of the IQOLA project. A ‎sample of children completed the measure in a clinical setting, with a subgroup completing it ‎again to assess test–retest reliability. Concurrent criterion validity was evaluated by having all ‎participants complete the Modified Child Dental Anxiety Scale (MCDAS) alongside the ‎CEDAM. Construct validity was examined using exploratory and confirmatory factor ‎analyses.‎

**Results:**

The study included 275 children between the ages of 9 and 16. The Iranian version of CEDAM ‎exhibited excellent internal consistency with a Cronbach's *⍺* coefficient of 0.83. Test–retest ‎reliability was also high, with an intraclass correlation coefficient value of 0.96. Furthermore, there was a significant and ‎positive correlation between CEDAM and MCDAS scores (*ρ* = 0.72, *p* < .01). Exploratory ‎factor analysis identified two factors, and confirmatory factor analysis confirmed that the ‎instrument aligned well with the factor structure obtained from the exploratory analysis.‎

**Conclusion:**

This study provides evidence supporting the validity and reliability of the Iranian version of ‎CEDAM as a valuable tool for evaluating dental anxiety in Persian‐speaking children between ‎the ages of 9 and 16.‎

## 1 INTRODUCTION

Dental fear and dental anxiety are common experiences for children and adolescents‎‏ ‏and have ‎been recognized in many countries as a public health dilemma (Shim et al., 2015). Dental ‎fear is a normal emotional reaction that individuals experience in response to specific ‎threatening stimuli encountered during dental procedures. Dental anxiety encompasses a state ‎of unease in which individuals anticipate negative experiences during dental procedures, often ‎accompanied by a perceived loss of control. These terms are often employed without ‎differentiation despite their nuanced distinctions in meaning (Klingberg & Broberg, 2007). ‎From a psychological perspective, dental fear primarily develops during childhood and ‎adolescence (Porritt et al., 2012; Shim et al., 2015).‎

Even in mild forms, dental anxiety can significantly contribute to behavioral management challenges in children (Klingberg & Broberg, 2007). When children encounter new stimuli, experience aversive sensations like pain or discomfort, or perceive potential threats without an opportunity to escape, they are more likely to exhibit uncooperative behaviors (Coxon, 2019). Children experiencing dental fear and dental anxiety often tend to avoid seeking professional dental care, which can have detrimental effects on their oral health. This avoidance may result in deteriorated oral health conditions, delayed or emergency treatments, and an increased likelihood of requiring more extensive procedures like tooth extractions (Milgrom et al., 1995). Furthermore, their uncooperative behavior while in the dental chair adds to the job‐related stress experienced by dental professionals (Bankole et al., 2002; Crego et al., 2014).

Research on the prevalence of dental fear in children has been conducted extensively across various cultures and socioeconomic groups. However, despite the considerable number of studies, the reported figures exhibit a wide range, making it challenging even for structured review articles to establish a more precise and narrow prevalence range. As an example, in a review of 12 populations, Klingberg and Broberg (2007) reported a wide prevalence range of dental fear, ranging from 6% to 20%. The wide range can be attributed to various factors, including cultural norms and their influence on individual psychological patterns, differences in sampling methods, and variations in the measurement techniques used to assess dental anxiety.

A key aspect in the dental care of pediatric patients is the effective management of their behavior, particularly in cases where anxiety is present. Dental fear acts as a psychological barrier, underscoring the importance for dentists to acquire comprehensive knowledge in understanding and addressing this emotion conceptually and operationally. By proactively identifying fear early in the dental visit and tailoring treatment plans accordingly, dentists can better cater to the needs of anxious patients (Cianetti et al., 2017).

To identify patients experiencing dental anxiety, various methods have been developed, including the utilization of the Frankl scale to evaluate behavioral responses during dental visits. Other methods involve measuring physiological indicators such as pulse, blood pressure, and muscular tension, in addition to employing psychometric scales (Cázares de León et al., 2020).‎ Two widely recognized measures for assessing dental anxiety in children are the Children's Fear Survey Schedule‐Dental Subscale (CFSS‐DS) (Cuthbert & Melamed, 1982) and the Modified Child Dental Anxiety Scale (MCDAS) (Humphris et al., 1998). These measures have demonstrated reliability and validity across multiple countries. However, it is important to note that they may present challenges for children who are visiting the dentist for the first time as they inquire about specific dental and medical situations. Additionally, these measures do not assess the mental and emotional patterns associated with dental anxiety (Porritt et al., 2013). ‎

The Children's Experience of Dental Anxiety Measure (CEDAM) is a new measure introduced in 2018, specifically designed to gain insights into the psychological patterns of dental anxiety in children. The CEDAM employs a 14‐question scale based on the Five Areas cognitive behavioral theory (CBT) model, aiming to capture the child's thoughts, feelings/physical symptoms, and behaviors related to dental visits. Unlike other measures, the CEDAM does not focus on specific dental situations, making it more suitable for children who are visiting the dentist for the first time. By examining the child's thoughts and feelings associated with dental anxiety, the CEDAM assists dental healthcare workers and parents in effectively managing the situation and reducing the child's anxiety levels. It has been reported as a valid and reliable tool for assessing children's dental anxiety (Porritt et al., 2018).

‎There is an increasing acknowledgment of the need to prioritize psychological interventions aimed at effectively reducing long‐term dental anxiety among patients (McGoldrick et al., 2001). Guided self‐help CBT offers a highly effective and child‐centered approach to alleviating child dental anxiety by enhancing patients' coping skills (Shim et al., 2015). The unique attributes of the CEDAM enable it to discern the internal aspects of dental anxiety experienced by children, as well as the underlying factors that contribute to its persistence. Consequently, the CEDAM serves as a valuable tool for identifying specific areas that necessitate intervention and can be targeted for effective change in reducing the child's anxiety (Bux et al., 2019). The use of CEDAM has facilitated the evaluation of changes in dental anxiety during the course of dental treatment, specifically when children have utilized the guided self‐help resource (Porritt et al., 2018). Additionally, its suitability extends beyond research, encompassing clinical settings and healthcare evaluations (Bux et al., 2019). ‎

To ensure the suitability of a measure for use in different countries, it is crucial to translate it in a manner that aligns with their language structure and cultural context (Gandek & Ware, 1998; Ware et al., 1995). Iranian versions of CFSS‐DS and MCDAS have been developed and reported to be highly valid and reliable in assessing the dental anxiety of children (Javadinejad & Farajzadegan, 2013; Safari et al., 2018). However, these measures still have certain limitations, as mentioned earlier. Given the high prevalence of dental anxiety in the Iranian population (Paryab & Hosseinbor, 2013; Salem et al., 2012; Zarafshan et al., 2015) and its significant impact on oral health (Crego et al., 2014; Lin, 2013), the development of a measure that effectively identifies dentally anxious patients and is culturally suitable for the Persian‐speaking community becomes highly important.

Given that the CEDAM has not yet been utilized in non‐English‐speaking populations and the absence of normative data for the Iranian context, this study sought to develop and assess the validity and reliability of the Iranian version of the CEDAM. The aim was to employ confirmatory factor analysis (CFA) to establish a robust tool that can be used for clinical and research purposes in the future.

## 2 MATERIALS AND METHODS

### 2.1 Design and participants

**Figure 1:**
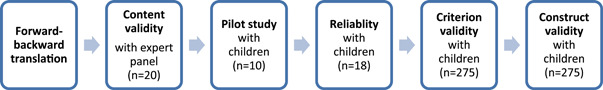
Flowchart showing an overview of the study strategy.

This research involved a two‐stage cross‐sectional descriptive–analytical study. In the first stage, the Iranian version of the CEDAM was developed, and its content validity was assessed. The second stage focused on evaluating the reliability, criterion validity, and construct validity of the CEDAM (as shown in Figure 1).

Following the approval of the National Committee for Ethics in Biomedical Research (Isfahan University of Medical Sciences) with the ethics code IR.MUI.RESEARCH.REC.1398.071, this study was conducted at the Dental School of Isfahan University of Medical Sciences and its affiliated dental clinics and included the children aged between 9 and 16 years who visited these facilities during April and May of 2019. Participation in the study was voluntary, and written consent from parents and oral consent from the children were obtained before enrollment. The inclusion criteria included being Persian‐speaking, aged between 9 and 16 years, and expressing a willingness to participate. Patients with conditions such as swelling, trauma, systemic illnesses, or severe disabilities were excluded from the study.

Demographic information of the participants, including gender, age, living conditions, number of siblings, birth order, and history of previous dental visits, was recorded for analysis.

### 2.2 Stage 1: Development of the Iranian version of the CEDAM and content validity assessment

#### 2.2.1 Overview of the original measure

The original English version of the CEDAM consists of 14 questions using a three‐point Likert scale, representing low anxiety (one point), moderate anxiety (two points), and severe anxiety (three points). The overall score, ranging from 14 to 42, represents the sum of points for all items and indicates the level of dental anxiety, with lower scores indicating lower anxiety levels (Porritt et al., 2018).

#### 2.2.2 Translation process

The translation of the CEDAM into Persian followed the IQOLA project guidelines (Bullinger et al., 1998) and consisted of two steps. In the first step, two independent Persian‐speaking translators translated the measure from English to Persian (forward translation). The second step involved translating the Persian version back into English (backward translation). The English version was compared to the original measure to ensure conceptual similarity and any necessary adjustments were made to align the meaning and concept with the original version.

#### 2.2.3 Content validity

Content validity assesses a measure's ability to fully cover the range of the construct it aims to measure. In this study, a panel of 20 experts, including pedodontists, child psychologists, oral public health experts, general dentists, and an expert in the development of quality‐of‐life assessment measures, evaluated each item of the CEDAM for simplicity, clarity, relevance, and essentiality. The content validity ratio (CVR) and content validity index (CVI) were calculated for each item. Based on Lawshe's table, items with a CVR score of ≥0.42 were considered essential. Items with CVI scores of ≥0.79 were considered acceptable, while those with a CVI score of 0.7‒0.79 were corrected, and those with a CVI score <0.7 were eliminated from the measure (Lawshe, 1975). By applying these criteria, the final Iranian version of the CEDAM included only items that demonstrated acceptable CVR and CVI scores.

#### 2.2.4 Pilot study

The pilot testing of the Iranian version of the CEDAM was conducted on a sample of 10 children who met the inclusion criteria. This step aimed to assess the children's understanding of the measure and identify any areas that required further refinement. Based on the feedback received during the pilot testing, final adjustments were made to the measure.

### 2.3 Stage 2: Evaluation of reliability, criterion validity, and construct validity

According to Mundfrom et al. (2005), the sample size for conducting a factor analysis should be determined based on the number of items in the questionnaire. In this study, the final Persian questionnaire consisted of 13 items. Following the guideline of having a minimum of 20 individuals per questionnaire item and accounting for potential data loss, the total sample size was determined to be 275 participants.

During this stage, the participants completed both the CEDAM and MCDAS questionnaires independently. However, a researcher was present during the process to offer assistance or address any questions that the children may have had.

#### 2.3.1 Reliability

Internal consistency, which refers to the extent to which items on a measure assess the same construct, was evaluated using Cronbach's *⍺* test. Additionally, test–retest reliability was assessed to examine the stability of the scores over time. A subgroup of 18 children, who were different from the main sample, completed the CEDAM again after a 2‐week interval. The intraclass correlation coefficient (ICC) was calculated to determine the agreement between the baseline and retest scores (Bujang & Baharum, 2017).

#### 2.3.2 Criterion validity

Criterion validity involves assessing the relationship between the scores obtained from a measure and an established outcome. Concurrent validity, a type of criterion validity, compares the ability of a measure to assess a construct with an existing gold standard. In this study, concurrent validity was examined by administering both the Iranian version of the MCDAS and the CEDAM to all participants simultaneously. The correlation between the overall scores of these two instruments was analyzed using Spearman's correlation coefficient.

#### 2.3.3 Construct validity

Construct validity refers to the extent to which a test accurately measures the intended construct. In this study, construct validity was evaluated by randomly dividing the data into two groups: one group with 140 samples and another with 135 samples. The first group, containing 140 samples, was used for exploratory factor analysis (EFA). EFA was performed on the first half of the sample (training sample) based on the principal components analysis extraction approach for estimating the factor loadings and the orthogonal Varimax Rotation to interpret the extracted factors. Factors were retained for further analysis based on the eigenvalues and Scree plot. Factor‐item loading values > 0.30 and factors with eigenvalues >  1 were considered as cutoffs to ensure more interpretable factors and explain sufficient amounts of the overall variation. Kaiser–Meyer–Olkin (KMO) measure of sample adequacy (values > 0.7) and Bartlett's test of sphericity (*p* <  .05) were used to determine data viability for factorability.

Factors obtained from EFA were used to perform CFA on the second group containing 135 samples. A minimum of five samples per item are needed for CFA (Greiff & Heene, 2017), and thus, the second group of 135 samples was considered adequate for CFA. CFA was evaluated using the comparative fit index (CFI), minimum discrepancy divided by its degree of freedom (CMIN/DF), and root mean square error of approximation (RMSEA) indices.

#### 2.3.4 Statistical analyses

The data were analyzed using IBM SPSS 23 (IBM Corp., Armonk, NY, USA), and the CFA was conducted using AMOS 24 (Arbuckle, J. L. Chicago: IBM SPSS).

## 3 RESULTS

### 3.1 Dental anxiety scores and study population demographics

Among the 275 participants (mean age = 11.67, SD = 1.96), 146 (53%) were male, and 129 (47%) were female. The mean CEDAM score for the entire population was 20.05 (SD = 4.8), and the mean MCDAS score was 17.26 (SD = 6.13). There were no significant differences in dental anxiety scores based on age, gender, living arrangement, or birth order, as determined by independent‐sample *t*‐tests and one‐way analysis of variance (*p* > .05). However, a correlation was found between dental anxiety scores and previous dental visits (*p* < .01). Furthermore, a post hoc analysis revealed that participants who had visited the dentist more than twice before the study had a significantly lower CEDAM dental anxiety score compared to others (*p* < .01). Additionally, children with more than two previous dental visits had significantly lower MCDAS dental anxiety scores than those who had never visited the dentist before (*p* < .01). Mean CEDAM and MCDAS scores for each demographic characteristic are summarized in Table 1.

**Table 1:** Mean CEDAM and MCDAS dental anxiety scores according to participants' demographic characteristics.

	*N*	%	CEDAM			MCDAS		
			Mean	SD	*p* Value	Mean	SD	*p* Value
Total	275		20.05	4.80	‐	17.26	6.13	‐
Sex								
Male	146	53	19.6	4.79	.07	16.8	6.34	.1
Female	129	47	20.6	4.78		18	5.85	
Age								
9–12	182	66	20.4	4.77	.1	17.6	6.26	.33
13–16	93	34	19.4	4.82		16.9	5.88	
Living arrangement								
Cohabiting parents	262	95	20	4.75	.28	17.2	6.07	.15
Single parent/other caregivers	13	5	21.5	5.82		19.8	7.13	
Order of birth								
Single/first child	126	54	19.5	5.04	.12	16.7	6.16	.20
Second/third/fourth	94	46	20.6	4.89		17.8	6.20	
Number of siblings								
0	63	23	20	5.37	.46	17.9	6.90	.95
1	116	42	19.5	4.50		16.6	5.87	
≥2	75	30	20.4	4.60		17.2	5.78	
Previous visits to the dentist								
0 (first time)	22	8	23.13	3.37	<.01	21.95	5.26	<.01
1	39	14	21.48	4.50		18.23	5.98	
2	62	23	21.08	4.48		18.31	5.60	
≥3	152	55	18.82	4.82		16.08	6.12	

*Note*: Because of missing values, in some groups, the sum does not add up to the total.

Abbreviations: CEDAM, Children's Experiences of Dental Anxiety Measure; MCDAS, Modified Child Dental Anxiety Scale; SD, standard deviation.

### 3.2 Content validity and pilot study

Item 7 on the CEDAM (think things could go wrong) had a CVI score of 0.64 and a CVR score of 0.2, leading to its removal from the Iranian version of the CEDAM. Items 4 (worry if need to have something done), 5 (think they will stop if asked), and 9 (feel shaky) had CVI scores of 0.72, 0.78, and 0.72, respectively, and were modified to ensure better comprehensibility in the Persian language for children. The remaining items obtained acceptable CVR and CVI scores, resulting in a final Iranian version of the CEDAM with 13 items. In the pilot study, minor adjustments were made to enhance the measure's clarity for children. The term “feeling” was removed from the last four items, and more fluent Persian equivalents were used. For instance, “feeling angry” was changed to “getting angry.” Table 2 presents a side‐by‐side comparison of the original version of CEDAM and the finalized ‎Iranian version.‎

**Table 2:** Original and Iranian versions of the CEDAM questionnaire.

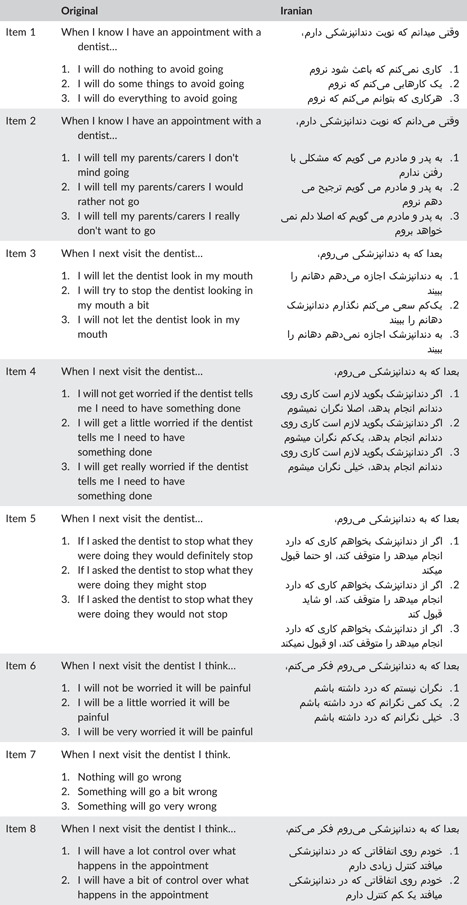
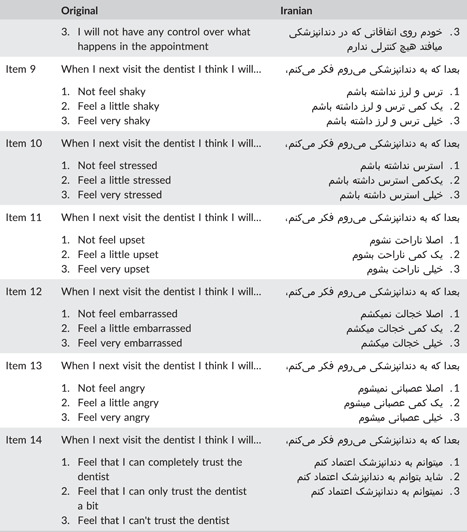

Abbreviation: CEDAM, Children's Experiences of Dental Anxiety Measure.

### 3.3 Reliability

A good internal consistency was demonstrated by calculating Cronbach's *⍺* for the CEDAM and MCDAS (0.83 and 0.86, respectively). Both the CEDAM *and* MCDAS exhibited excellent test–retest reliability with a 2‐week retest period (ICC = 0.968 and 0.983, respectively, *p* < .01).

### 3.4 Concurrent validity

The computation of Spearman's rank correlation coefficient revealed a high correlation between CEDAM and MCDAS scores (*ρ* = 0.727, *p* < .01).

### 3.5 Construct validity

Two factors were identified through EFA with Varimax Rotation, accounting for 44% of the total variance with KMO = 0.825 (Table 3). Factor I, characterized by emotions and control over the treatment, accounted for 33% of the variance, while Factor II, fear of the treatment, accounted for 11% of the variance. Fit indices in CFA showed a good fit between the instrument and the model obtained from the EFA (CFI = 0.98, CMIN/DF = 1.69, RMSEA = 0.06).

**Table 3:** Rotated CEDAM factor matrix.

Item	Factor loading	
	Emotions/control over the treatment	Fear of the treatment
Being embarrassed	0.741	
Being able to trust the dentist	0.695	
Being upset	0.653	
Being angry	0.636	
Avoidance	0.510	0.410
Think they will stop if asked	0.503	
Think will have control over what happens	0.474	0.420
Let the dentist look in the mouth	0.436	
Think it will be painful		0.720
Being stressed		0.701
Being fearful		0.666
Talk to parents about whether they want to go		0.616
Worry if need to have something done		0.585
*Percentage of variance explained*	22.1	21.9
*Eigenvalue*	4.29	1.43

Abbreviation: CEDAM, Children's Experiences of Dental Anxiety Measure.

## 4 DISCUSSION

Dental anxiety is a commonly experienced issue that tends to develop predominantly during childhood and adolescence, although it can affect individuals of all age groups (Shim et al., 2015). Understanding the nature and levels of dental anxiety is crucial for effective management strategies, especially considering the influence of social and cultural norms on individuals' responses to stress and anxiety (Porritt et al., 2012). In the context of Iranian children, it is important to have a culturally sensitive and linguistically appropriate measure to assess dental anxiety accurately. The Iranian version of the CEDAM demonstrated its validity and reliability as a tool for effectively assessing dental anxiety in the children who participated in the present study.

During the translation and content validity evaluation, it was observed that the word “shaky” and the phrases “feeling angry, embarrassed, upset, and so forth” posed difficulties for Persian‐speaking children in understanding. To mitigate potential misunderstandings, the term “shaky” was replaced with “fearful,” and the phrases “feeling angry, embarrassed, upset, and so forth” were modified to ‘being angry, embarrassed, and so forth.” Furthermore, the phrase “something going wrong” was deemed incomprehensible for Persian speakers and obtained a low content validity score. As a result, it was excluded from the Iranian version of the CEDAM. These modifications aligned the wording of the CEDAM with the structure of the Persian language, ensuring that the final Iranian version of the CEDAM is easily understood by Persian speakers. Consequently, the final Iranian version of the CEDAM consisted of 13 items that assessed various aspects related to dental visits, such as the child's trust in the dentist, concerns about dental interventions, sense of control over treatment, worries about pain, and feelings of fear, stress, upset, anger, and embarrassment.

The Iranian version of the CEDAM demonstrated satisfactory internal consistency, as indicated by a Cronbach's *⍺* coefficient of 0.83, which is consistent with the findings of Porritt et al. (2018) regarding the English version of the CEDAM. Similarly, the Iranian version of the MCDAS, developed by Javadinejad and Farajzadegan (2013), exhibited good internal consistency with a Cronbach's *⍺* of 0.86 in this study. These results indicate that the Iranian versions of both the CEDAM and MCDAS possess comparable internal consistency, suggesting the uniformity of the CEDAM. Furthermore, the test–retest reliability of both Iranian versions of the CEDAM and MCDAS demonstrated excellent consistency over time, reinforcing their reliability as measures.

Despite the CEDAM primarily addressing the emotional and cognitive aspects of dental anxiety (Porritt et al., 2018), while the MCDAS specifically targets dental procedures and situations, a notable correlation was found between the scores of their Iranian versions. This significant and positive correlation indicates the strong criterion validity of the Iranian version of the CEDAM in assessing dental anxiety.

Two factors emerged from the EFA of the Iranian version of the CEDAM, namely, “emotions and control over the treatment” and “fear of the treatment.” The observed factor loading pattern may be influenced by cultural values. Notably, two items, “avoiding the appointment” and “control over what happens during the appointment” on the Iranian version of the CEDAM, were loaded (≥0.4) on both factors, indicating a potential correlation between the extracted factors. The satisfactory fit obtained from CFA between the measure and the two‐factor model analysis resulting from EFA supports the construct validity of the Iranian version of the CEDAM. The English version of the CEDAM has also demonstrated good construct validity, albeit through the “known group” method (Porritt et al., 2018). Further studies, preferably with larger sample sizes, are warranted to better evaluate the factor analysis of the CEDAM.

Considering the adjustment in the range of anxiety scores between the English and Iranian versions of the CEDAM due to the removal of one item in the Iranian version, the mean anxiety score obtained from the Iranian version of the CEDAM in this study (20.05) closely approximates the mean score reported in the English CEDAM (21.09) by Porritt et al. (2018).

No significant differences in anxiety levels were observed between the Iranian versions of CEDAM and MCDAS across gender, age, number of siblings, order of birth, and living arrangement. However, the number of previous visits to the dentist emerged as a significant factor influencing children's anxiety levels, indicating that children who had visited the dentist more than twice exhibited lower levels of anxiety compared to those with fewer dental visits. Paglia et al. (2017) achieved similar results with the MCDAS and CFSS‐DS in the Italian population but reported higher dental anxiety levels in children aged 4‒7 than their 8‒11‐year‐old counterparts. The study conducted by Armfield et al. (2006) examined dental fear in a large sample of Australian ‎participants. They categorized participants into different age groups and found that individuals ‎aged 40–64 years reported the highest levels of dental fear. Females exhibited higher levels of ‎dental fear than males. Furthermore, participants who had not visited a dentist for more than 10 ‎years reported greater dental fear compared to those who had a dental visit within the previous ‎‎12 months‎ (Armfield et al., 2006). Porritt et al. (2018) did not observe any significant associations between age and gender with CEDAM scores, indicating that these factors did not influence dental anxiety levels. However, they did find that girls had higher MCDAS scores, suggesting a higher level of dental anxiety in female participants.

One study conducted on an Iranian population by Nilchiyan and Mohammadi (2013) reported higher levels of anxiety in girls using a pictorial version of MCDAS. Additionally, Safari et al. (2018) found lower anxiety levels in children who had previously visited the dentist compared to those who were visiting for the first time using the CFSS‐DS. Similarly, Mohebbi et al. (2019) assessed the CFSS‐DS in children aged 7‒11 and found higher dental fear in children without a history of dental visits. In contrast, Mohammadi et al. (2019) reported lower anxiety levels in 15‒16‐year‐olds without a history of dental visits compared to those who had recently visited a dentist in an Iranian population.

It is important to note that apart from the original study introducing the CEDAM and the present study, no other research has examined the CEDAM. Therefore, there is limited data available for comparison of CEDAM scores. Furthermore, it is worth mentioning that the current study did not assess the responsiveness of the Iranian version of the CEDAM to changes, highlighting the need for further research in this area.

The utilization of the CEDAM in clinical practice can serve as a valuable tool for enhancing communication and gaining insights into patients' concerns and worries regarding dental treatment. By assessing CEDAM scores, dental teams can identify factors contributing to patients' anxiety and develop tailored interventions. For instance, if a child reports low perceived control, the team can offer support and implement strategies to enhance the child's sense of control. Similarly, if physiological symptoms of anxiety are present, the team can focus on teaching relaxation techniques (Porritt et al., 2021). The CBT assessment model, utilized in the development of the measure, seeks to provide ‎clinicians and patients with valuable information regarding the specific factors that can be ‎addressed to alleviate anxiety (Williams & Garland, 2002). Additionally, the CEDAM can be used to monitor changes in dental anxiety over time and assess the effectiveness of interventions. It is worth noting that the act of communicating anxiety to the dental team itself can alleviate feelings of anxiety (Dailey et al., 2002).

In conclusion, this study demonstrates that the Iranian version of CEDAM is a valid and reliable ‎measure for assessing dental anxiety in Persian‐speaking children aged 9‒16.‎ The successful translation and validation of CEDAM in the Iranian context demonstrate its potential for cross‐cultural use. This study's findings suggest that the measure can be applied beyond its original cultural and linguistic context, providing valuable insights into dental anxiety in diverse populations. The availability of a validated tool like CEDAM enables clinicians and researchers in Iran to effectively assess and monitor dental anxiety in children. By identifying children with high dental anxiety, dental professionals can customize interventions and strategies to provide appropriate support and reduce anxiety during dental visits. This study contributes to the existing research on dental anxiety measurement and expands the range of available instruments for researchers studying dental anxiety in children. The validation of the Iranian version of CEDAM allows for further investigation into dental anxiety prevalence, risk factors, and treatment outcomes within the Iranian population.

## AUTHOR CONTRIBUTIONS

Zahra Enshaei and Kimia Sadeghi Kaji conceived the ideas. Kimia Sadeghi Kaji and Zahra Saied‐Moallemi collected and analyzed the data. Zahra Enshaei, Zahra Saied‐Moallemi, and Kimia Sadeghi Kaji led the writing.

## CONFLICT OF INTEREST STATEMENT

The authors declare no conflict of interest.

## Data Availability

The data that support the findings of this study are available from the Vice‐Chancellery for Research and ‎Technology, Isfahan University of Medical Sciences. Restrictions apply to the availability of these data, ‎which were used under license for this study. Data are available from the authors with the permission ‎of the Vice‐Chancellery for Research and Technology, Isfahan University of Medical Sciences.‎
